# An e-registry for household contacts exposed to multidrug resistant TB in Mongolia

**DOI:** 10.1186/s12911-020-01204-z

**Published:** 2020-08-12

**Authors:** Kush Naker, Katherine M. Gaskell, Munhjargal Dorjravdan, Naranzul Dambaa, Chrissy H. Roberts, David A. J. Moore

**Affiliations:** 1 London School of Hygiene and Tropical Medicine, Keppel Street, London, WC1E 7HT UK; 2 National Centre of Communicable Diseases, Nam Yan Zhu Street, 13th Khoroo, Ulaanbaatar, Mongolia

**Keywords:** Multidrug-resistant tuberculosis, Household contacts, Registry, Electronic

## Abstract

**Background:**

The WHO recommends that individuals exposed to persons with multidrug resistant tuberculosis (MDRTB) should be screened for active TB and followed up for 2 years to detect and treat secondary cases early. Resource prioritisation means this is rarely undertaken and where it is performed it’s usually using a paper-based record, without collation of data. Electronic data collection into a web-based registry offers the opportunity for simplified and systematic TB contact surveillance with automatic synthesis of data at local, regional and national level. This pilot study was designed to explore the feasibility of usage of a novel e-registry tool and explore obstacles and facilitating factors to implementation.

**Methods:**

In parallel with their paper records, seven dispensaries in Ulaanbaatar, Mongolia collected standardized data electronically using Open Data Kit (ODK). Patients with MDRTB and their contacts were recruited during a single clinic visit. Staff and patients were interviewed to gain insights into acceptability and to identify areas for improvement.

**Results:**

Seventy household contacts of 32 MDR-TB index patients were recruited. 7/70 contacts (10%) traced had active TB at the time they were recruited to the e-registry.

Paper registry satisfaction was low; 88% of staff preferred the e-registry as it was perceived as faster and more secure. Patients and their contacts were generally supportive of the e-registry; however, a significant minority 10/42 (24%) of index cases who were invited, declined to participate in the e-registry, with data security cited as their top concern.

**Conclusion:**

E-registries are a promising tool for MDRTB contact tracing, but their acceptability amongst patients should not be taken for granted.

## Background

Despite having been recommended by the World Health Organization (WHO) since 2006, and repeatedly thereafter [1–6], contact tracing for multidrug resistant tuberculosis (MDRTB), remains a low priority within national TB programs in many low and middle-income countries where TB burden is high. In 2017, only a quarter of the estimated 558,000 rifampicin resistant and multidrug resistant tuberculosis (RR/MDRTB) cases worldwide were enrolled on effective treatment, with many dying even before a diagnosis could be made [7]. Significant improvement in MDRTB case ascertainment is essential to meet the END-TB strategy targets on reducing TB incidence and mortality [6, 7]. Observational data in support of the need to provide treatment for latent TB infection (LTBI) presumed to be MDR is gradually accumulating [8, 9] and three randomised controlled trials of preventive therapy for MDR exposed contacts are now underway [10–12].

Meta analyses of MDRTB contact tracing studies have shown that 41.3–61.3% of household contacts have latent TB infection, and that 3.4–6.5% develop active disease [13–15]. Despite significant heterogeneity in the studies included in these systematic reviews they have consistently identified a substantial yield of secondary tuberculosis cases among household and close contacts. Most secondary cases tested also had MDR and were diagnosed shortly after the index case [14].

The WHO has developed a set of global recommendations for TB contact investigation and these encourage the use of a set of standardised approaches to programme monitoring and evaluation. Heterogeneity of data collection between centres and of clinical definitions in datasets collected by various agencies remain a barrier to effective global monitoring. Whilst the WHO guidelines include precise definitions of index cases, close contacts and household contacts [5, 16, 17], a minimum dataset for an MDRTB contact registry has yet to be internationally agreed [9, 18] and no agency currently provides support in the form of data collection tools through which such a dataset could be uniformly collected, aggregated, analysed and disseminated.

A variety of electronic TB registries and surveillance systems have been reported in recent years, including ETR.net (www.etrnet.info), ENRS (ccs.gov.eg/ntp/M_E_ENRS.htm), and e-TB manager (etbmanager.org/) [19, 20].

Open Data Kit (ODK, https://opendatakit.org) is a free open source data collection toolkit allowing developers to design forms for an android application (app) ODK collect. Working in Botswana, Ha and colleagues developed an electronic data collection (EDC) solution based on ODK and used this to facilitate screening of TB contacts during household visits [21]. They concluded that using ODK reduced the time taken to complete tracing for each contact and scored favourably for user satisfaction among the health workers who conducted the tracing [21].

Paper based methods remain the standard approach in those few countries which currently undertake any MDRTB contact tracing with or without subsequent surveillance. Very few studies have evaluated the feasibility and acceptability of EDC tools in these countries [22]. Where these have been addressed, data accuracy and completeness remain an issue and none of this work has been completed in MDRTB contact tracing [23, 24]. In this study we aimed to evaluate the feasibility of a novel mobile electronic data collection tool for the building of a web-based MDRTB contact e-registry, and we collected descriptive epidemiological data on contacts.

The study was carried out in Mongolia, where in 2017 the estimated incidence rate then was 428/100,000 with a TB disease notification rate of 136/100,000, and a case detection rate of 32% [25]. 12.6% of laboratory confirmed cases were MDR [26]. MDRTB contact tracing has been national policy since 2006, though implementation is patchy. In 2016, 5.7% of identified household contacts developed active TB [26]. Contact tracing is done through district dispensaries where patients receive directly observed treatment (DOT).

## Methods

### Ethics approval and consent to participate

The study was approved by the Mongolian Ministry of Health research ethics committee (Ref: 18) and the London School of Hygiene and Tropical Medicine (Ref: 13882).

### Consent

All participants (staff and patients) were provided written information about the research and gave written informed consent to participate, as well as to having anonymised data shared. For children below 18 years of age, consent was taken from a parent in addition to consent from the child participant. Patients who chose not to participate in the e-registry were also invited to complete an optional anonymous feedback form to explain their reasoning.

### Study site selection

Seven dispensaries were selected to participate based on their case load and physical proximity. At the time of the study 283 patients were receiving treatment for MDR-TB across these sites, these accounted for 57% of the national MDR-TB cases [26]. They covered six districts, all within Ulaanbaatar city limits, facilitating access for the investigators to as many sites as possible within the limited time frame of the study.

### Participant selection and recruitment

All staff whose role already involved contact tracing at the selected dispensaries were invited to participate in the study. Index patients were invited to participate through convenience sampling if they attended any of the study sites over the 3 week study period in August–September 2017, and met the following inclusion criteria; microbiologically confirmed MDR-TB (pulmonary and/or extrapulmonary) and capacity to consent. Patients newly diagnosed during the study period, as well as those already undergoing treatment were eligible. These index patients were then asked to invite their household and close contacts as defined below to attend the dispensary for recruitment, who were eligible if they too had capacity to consent. Patients were excluded from recruitment if they lacked capacity to consent, and index patients were excluded if they had a drug susceptibility pattern not consistent with MDR-TB as defined by the WHO.

### Data collection

Open Data Kit (ODK) was chosen as a convenient platform for the e-registry as it is an open access software tool used widely by LSHTM in research and outbreak response, making it an attractive option for a project with limited funds and meant it could be set up rapidly. Data is entered into the ODK collect application on android tablets. The data is hosted on ODK Aggregate V ≥ 1.4.15 (opendatakit.org) running on a mirrored 256-bit encrypted Apache Tomcat server with a MySQL back-end provided in a dual virtual server configuration protected by an enterprise firewall and hardware load balancer. Both servers are replicated every 2 hours, further backed up daily and back up data is streamed to tape weekly. Both servers are also automatically patched with the latest security updates.

Personal identifiable information was first collected on a registration form which was encrypted before transmission to the server, remaining encrypted until downloaded and decrypted with a 2048-bit RSA key. Once data collection forms were marked as complete, they were no longer accessible through the ODK collect application, but the data could be accessed by the National Centre of Communicable Diseases (NCCD) on the ODK aggregate server, decrypted locally and emailed back to clinics on a scheduled basis in PDF or XLS format with end to end encryption. Patient data was not then available to view on the tablet.

For this study each dispensary was issued with an android tablet preloaded with the data collection forms on ODK collect. Local procurement of tablets or the use of staffs personal devices would also be feasible as the ODK data collections forms are downloaded to each device from the aggregate server. All dispensaries were already equipped with wired internet access, but WiFi routers were installed to allow the tablets internet access on-site. Security locks and applock software were installed on the tablets to prevent unauthorised access as well as restricting the use of the tablets. All dispensary staff had prior computer literacy with experience in using a PC for electronic data capture, as well as personal access to smartphones. However, none had reported experience of electronic data capture with a tablet computer. All staff were trained to recruit and consent participants, operate the tablets and app, and provided with a written manual and contact details for technical support. Staff continued recording all contact tracing activities on the paper-based registries during the study. Data was extracted into Microsoft Excel 2016 to calculate interquartile range (IQR).

### Acceptability

All staff who participated in the study underwent a semi-structured interview after using ODK for at least 2 weeks. Patient participants and non-participants were asked to fill out an anonymous written feedback questionnaire to determine acceptability. Responses to closed questions were reported descriptively. Responses to open questions were coded and themes were identified using framework analysis [27].

### Definitions

The definitions for the index case and household contact used 2012 WHO recommendations [5], the definition for close contact was based on the Mongolian tuberculosis program guidelines, which uses a higher threshold than the WHO [28].

Index case: The initially identified patient with new or recurrent TB in a specific household or comparable setting in which others may have been exposed. Note: The index case is the initial patient accessing healthcare, they may not be the source case.

Household contact: A person who shared the same enclosed living space for ≥1 nights or for frequent or extended periods during the day with the index case during the 3 months before commencement of the current treatment episode.

Close contact: A person who is not in the household but shares an enclosed space with the index case, such as a place of social gathering, workplace, or facility, for greater than 40 h per week during the 3 months before commencement of the current treatment episode.

## Results

### MDRTB contact tracing in Ulaanbaatar

32 of 42 invited index patients with laboratory confirmed MDRTB agreed to participate in electronic registration of themselves and their contacts. The median age of index cases was 32.5 years, 21 were female. 27 had pulmonary disease, 4 had extra-pulmonary disease, and 1 had both. All 24 who were tested for HIV were seronegative. The median time from diagnosis to recruitment in this study was 15 months (IQR 6-20 months).

The index cases reported a total of 72 household contacts (HHC) and a further 123 close contacts. HHC were only recruited from consented index patients. Each index case had a median of 3 HHC (IQR 1–3, Range 0–6) and 1 close contact (IQR 0–5.5, Range 0–30). 70 of these contacts (69 household, 1 close) attended a dispensary and agreed to participate in the study.

Demographic data and investigation results were available on 68/70 contacts. The median age was 19.5 (IQR 6.75–35, range 1–57) and 35 (51%) were female. 12/68 (18%) contacts were aged under five, 5 had a low BMI, and 1 had a fibrotic lung lesion. 25 were HIV tested and all of these were negative. Nine contacts had symptoms suggestive of TB, 7 reported a cough lasting over 2 weeks, 6 reported fever, 5 reported weight loss and chest pain, 4 reported fatigue and sweats, and 1 reported shortness of breath. 59/68 (87%) had a chest x-ray, 10 of which were abnormal. 7/70 (10%) of contacts screened had laboratory confirmed secondary cases of active TB among six households (Table 1). Six had MDRTB and one had drug susceptible (DS) TB. Complete drug susceptibility testing (DST) was available in four secondary MDRTB cases, three of which had an identical profile to their index. 28 contacts underwent LTBI testing, 13/26 were TST positive, 4 had IGRA testing, all of whom were negative, 7 received TB preventive therapy.

**Table 1:** Characteristics of secondary TB cases

#	Age, gender and exposure Intensity	Symptoms	Chest X-ray	TST	Diagnosis
**1**	23 F, shares a bed	Cough, fever, chest pain, dyspnoea, fatigue	Abnormal	N/A	Sm + MDR (Gene Xpert) No DST
**4**	5 M, household	Cough	Abnormal	Positive	MDR Discordant DST
**11**	6 F, shares a bed	Nil	Abnormal	Positive	MDR Identical DST
**15**	21 M, shares bedroom	Nil	Abnormal	N/A	Sm + MDR Identical DST
**16**	14 M, shares bedroom	Weight loss	Abnormal	Positive	Sm- MDR (Gene Xpert) No DST
**37**	3 F, shares a bed	Cough, fever, weight loss, sweats, chest pain, fatigue	Abnormal	N/A	Sm + MDR Identical DST
**67**	46 M, household	Cough, fever, chest pain	Normal	N/A	Sm- DS-TB

#: Number, *DSTB* Drug susceptible tuberculosis, *DST* Drug susceptibility testing, *F* Female, *M* Male, *MDR* Multidrug resistant, *N/A* Not applicable, Sm+: Sputum smear positive, Sm-: Sputum smear negative

**Table 2:** Themes identified from staff interviews and anonymous feedback forms

**Advantages of ODK** • Records were less likely to be lost, particularly investigation results • Digital photos of x-rays could be stored for comparison over time • Easier to transfer contact tracing information for patients moving districts • Faster than paper, especially when using predictive text	**Disadvantages of ODK** • Submitted data was not immediately visible on the device • Registry numbers had to be entered multiple times (once on each form)
**Improvements suggested by staff** • Make data more rapidly available for viewing • Reduce the number of forms • Allow data entry on PC • Auto-populate certain fields e.g. follow up date • Create a system that would automatically send out reminders to patients via SMS/email of upcoming appointments	**Reasons given to staff by patients for refusing to join registry** • Already taking part in multiple other research studies • Didn’t have time to bring their contacts to dispensary during study period • Concern regarding security of their data being sent electronically – particularly that medical history would be shared on social media
**Improvements suggested by patient** • To be able see their own e-record.	**Reasons given on anonymous feedback forms** • Not wanting to share medical or personal data, even for research purposes • Concern that their ‘information would be lost in the internet’ • Not understanding the purpose of the study (non-participant)

### Staff satisfaction with e-registry system

8 of 12 trained staff involved in contact tracing during the study were interviewed (4 doctors, 3 nurses and 1 dietician). 4/8 reported being satisfied, and 3/8 reported being very dissatisfied with the paper-based registry. Staff reported it ‘takes a long time to find records, especially test results’ in the paper records system and that results paperwork and copies of x-rays were often kept by patients making it difficult to collate the data for quarterly reports, often duplicating work. All the staff reported being satisfied with the e-registry system, and the training received to use it. 4/8 staff stated a strong preference to use ODK for contact tracing, 3/8 slightly preferred ODK and one had no preference. All staff said they would be happy to continue using an e-registry and would recommend its expansion (Table 2).

### Patient satisfaction with e-registry system

Thirty-nine anonymous feedback forms were completed by index cases approached to be in the e-registry, 8/39 survey respondents did not consent to inclusion of their details in the e-registry. Of the 31 who agreed to have their details collected electronically 11 felt it worked very well, 19 slightly well, and 1 slightly poorly. 28/31 (90%) felt that the e-registry was better than paper, 2 didn’t know and one respondent left that question blank.

The most commonly cited concern, mentioned by 12 of the survey respondents, was the security of their data. 8 of these 12 were e-registry participants. This was closely followed by concerns that their data may be inadvertently ‘lost’, and two e-registry participants were unhappy about sharing information on their household income stating, ‘there is no need to ask about income’.

## Discussion

In this study we have developed and evaluated the feasibility and acceptability of an electronic MDRTB contact registry. A high yield of 10% (7/70) secondary TB cases in contacts reaffirms the importance of contact tracing and of a standardised and systematic approach to contact tracing in this setting.

Most of the data collected for the MDRTB contact registry overlaps with data collection necessary for the MDRTB index registry; a more unified system would therefore reduce duplication. Furthermore, many MDRTB patients have previously been treated for drug susceptible TB and using a common registration system across all types of TB disease could help prevent information on discrete sub-populations from forming data silos. Linking records across these different registries to avoid the duplication would be simpler in an electronic system and offer another advantage over paper.

There is a desire to improve the current MDRTB contact tracing and an e-registry is recognised as a way to achieve this by most patients and staff. However, refusal to participate in the e-registry by 10/42 (24%) of the index MDRTB cases invited does raise a concern regarding the acceptability of an e-registry hindering the contact tracing practices. Although not identified in the questionnaires, it is possible non-participation of indexes may have been influenced by the nature of presenting the e-registry as a research project co-ordinated by an external organisation, as opposed to paper registry which had been presented as being a routine part of their clinical management. As a result, this may have heightened the concerns regarding data security.

It is not possible for staff to view or search the registry from the ODK collect app, meaning they needed a separate way to access patient registry numbers, essential in linking the correct patient record. In effect, a second paper registry was made for the ODK registry to work. This duplication is inefficient and a potential source for error.

There is a clear need for an e-registry that provides a secure user interface. Two promising alternative systems are TUBIS and DHIS2, both are web based and allow the registry to be searched, simplifying it for users to retrieve patient records and add further data to the correct record. Each of the electronic registry systems mentioned above have their own relative merits and limitations [19]. WHO are now collaborating to provide support for TB patient data reporting using the DHIS2 platform. Inclusion of TB contacts in this software would be a logical next step [29, 30].

An internationally agreed minimum data set for contact tracing, and adherence to the shared definitions endorsed by the WHO would likely be more beneficial than standardised data collection tools to allow these to be more adaptable to local contexts.

### Study limitations

This study has several limitations. Firstly, staff being aware that they were taking part in a research study may have influenced the accuracy and completeness of data entry. Staff were not offered any incentives related to their performance, data accuracy or interview feedback. Conversely as the e-registry data was collected in duplicate with paper data, staff might have considered data accuracy for the e-registry to be less important.

Secondly, presenting the e-registry to patients as a novel tool under investigation as part of a research project, and requiring explicit informed written consent for their participation may have in and of itself heightened their anxiety regarding data security. The paper based registry in contrast was presented as the default option, with consent for data collection implied but not explicitly sought.

Thirdly, dispensary staff were aware that the interviewers had been involved in the development of the e-registry. This may have introduced response bias, skewing their interview answers to be more positive to be polite and avoid causing offence. They were asked to record an honest and open account of the experiences using the e-registry despite this.

Finally, all the dispensaries covered were urban or semi-urban, the results will not necessarily reflect experiences in rural areas.

## Conclusions

Deployment of an e-registry for MDRTB contact tracing is certainly technically feasible, and acceptable to the majority of patients and staff involved. Staff perceived benefits in reducing the likelihood of records being lost, being able to gather data more quickly and reducing the time taken to compile it for quarterly reports. However reluctance to participate in the e-registry amongst a significant minority of index patients could pose a major challenge in shifting to such systems in future, highlighting the importance of gathering patient feedback on the roll-out of such new systems.

A user-friendly secure e-registry within which TB staff can review patient management and provide reports, would enable care, follow-up and adherence to WHO guidance. E-registry systems should be explored for the use of MDRTB contact tracing and should seek to meet the following criteria:

- Accurate and secure – adequately backed up to prevent data loss and protected against unauthorised access
- User friendly – easy to learn, quick to use and unambiguous questions and responses presented in a logical manner
- Integrated into the TB and MDRTB case and contact registry systems
- Fully self-contained - not requiring any additional paper records to maintain the registry
- Capable of data entry offline
- Free and open source
- Accuracy of registry data should be audited periodically to ensure standards are being maintained
- A minimum dataset for MDRTB contact tracing needs to be internationally agreed
- Shared definitions for surveillance and research purposes need to be more widely embraced

## Supplementary information

**Additional file 1.** Index eregistry data.xlsx. Anonymsed eregistry data collected on index patients with MDRTB.

**Additional file 2.** Contact eregistry data.xlsx. Anonymised baseline characteristics and demographic data on eregistry for contacts.

**Additional file 3.** Contact eregistry test results data.xlsx. Anonmyised contacts data on follow up investigation results and outcomes.

**Additional file 4.** Interview Staff template V1 09042017.docx. Questionnaire template for staff interviews to assess acceptability of eregistry.

**Additional file 5.** Staff survey results.xlsx. Collated results of staff interviews conducted.

**Additional file 6.** Patient anonymous feedback V2 17,082,017.docx. Questionnaire template provided to patients for anonymous feedback on eregistry.

**Additional file 7.** Patient survey results.xlsx. Collated results of patients survey responses.

## Data Availability

The datasets generated and/or analysed during the current study are available in the Open Science Framework repository, available here: https://osf.io/n925x/ DOI: 10.17605/OSF.IO/N925X

## Abbreviations

DHIS2: District Health Information System 2
DOT: Directly observed treatment
EDC: Electronic data collection
IGRA: Interferon gamma release assay
IQR: Interquartile range
LTBI: Latent tuberculosis infection
MDRTB: Multidrug resistant tuberculosis
ODK: Open Data Kit
RR: Rifampicin resistant
TB: Tuberculosis
TST: Tuberculin skin test
TUBIS: Tuberculosis information system
WHO: World Health Organisation
