# Supplementary material for: An e-registry for household contacts exposed to multidrug resistant TB in Mongolia
Source: BMC Med Inform Decis Mak. 2020 Aug 12;20:188. doi: 10.1186/s12911-020-01204-z (PMC7425559; doi:10.1186/s12911-020-01204-z)
Supplement: Supplementary file 4 — Additional file 4. Interview Staff template V1 09042017.docx. Questionnaire template for staff interviews to assess acceptability of eregistry. [file 12911_2020_1204_MOESM4_ESM.docx]

**Participant ID:
Centre ID:**

**Role:**

**Date:**

**Do you own a smartphone or tablet? If yes, what make/model?**

**Have you previously encountered the use of an application for data entry for medical purposes?**

**How are you involved in MDR-TB contact tracing?**

**Could you describe the process in tracing and recording MDR-TB contacts - and along the way identify any positive of negative features of the process?**

Free response

Re-prompt identification of positive/negative features when describing documenting information for registry

**Is there a clear algorithm on how to screen contacts at each visit?**

**Are treatment plans consistent with the algorithm?**

**How satisfied are you overall with the current MDR-TB contact tracing procedures?**

Very Dissatisfied Somewhat dissatisfied Somewhat satisfied Very Satisfied

**How satisfied are you with the current PAPER BASED methods used to collect data for the MDR-TB contact registry?**

Very Dissatisfied Somewhat dissatisfied Somewhat satisfied Very Satisfied

**How satisfied are you with the ELECTRONIC methods used to collect data for the MDR-TB contact registry?**

Very Dissatisfied Somewhat dissatisfied Somewhat satisfied Very Satisfied

**How satisfied are you with the training received on using the electronic methods used to collect data for the MDR-TB contact registry?**

Very Dissatisfied Somewhat dissatisfied Somewhat satisfied Very Satisfied

**What could be improved with the training you received on how to use ODK?**

**How does ODK compare to the paper based methods for collecting data?**

Strongly prefer paper

Slightly prefer paper

Both Same

Slightly prefer ODK

Strongly prefer ODK

**What do you think are the advantages of ODK?**

**What do you think are the disadvantages of ODK?**

**Do you think ODK is faster, slower or the same as using paper?**

**How did you find the result feedback to your team?**

**Do you think ODK helped you to follow contact tracing protocols?**

**How many patients refused to use the app?**

**How would you feel about continuing using ODK in the future?**

**Would you recommend it to others and why?**

**How could ODK or an electronic data collection system be improved?**
