# Supplementary material for: An e-registry for household contacts exposed to multidrug resistant TB in Mongolia
Source: BMC Med Inform Decis Mak. 2020 Aug 12;20:188. doi: 10.1186/s12911-020-01204-z (PMC7425559; doi:10.1186/s12911-020-01204-z)
Supplement: Supplementary file 6 — Additional file 6. Patient anonymous feedback V2 17,082,017.docx. Questionnaire template provided to patients for anonymous feedback on eregistry. [file 12911_2020_1204_MOESM6_ESM.docx]

## **Feasibility of mobile electronic registry data collection for Multidrug Resistant Tuberculosis contacts in Mongolia**

**Anonymous Feedback**

1. Did you agree to having your information collected using the application on the tablet computer?
   Yes No
2. What were the reasons that made you say yes or no?
3. If you agreed, how well do you think it worked, and why?
   Very poorly Slightly poorly Slightly well Very well
4. If you agreed, do you have any concerns about being on the electronic registry?
5. Do you think online is better than paper
6. Can you think of any way the system could be made better in the future or comments?
